# Immune response to SARS-CoV-2 Omicron variant in patients and vaccinees following homologous and heterologous vaccinations

**DOI:** 10.1038/s42003-022-03849-0

**Published:** 2022-09-02

**Authors:** Claudia Maria Trombetta, Giulia Piccini, Giulio Pierleoni, Margherita Leonardi, Francesca Dapporto, Serena Marchi, Emanuele Andreano, Ida Paciello, Linda Benincasa, Piero Lovreglio, Nicola Buonvino, Nicola Decaro, Angela Stufano, Eleonora Lorusso, Emilio Bombardieri, Antonella Ruello, Simonetta Viviani, Rino Rappuoli, Eleonora Molesti, Alessandro Manenti, Emanuele Montomoli

**Affiliations:** 1 Department of Molecular and Developmental Medicine, University of Siena, Siena, Italy; 2 VisMederi srl, Siena, Italy; 3 VisMederi Research srl, Siena, Italy; 4 Monoclonal Antibody Discovery (MAD) Lab, Fondazione Toscana Life Sciences, Siena, Italy; 5 Interdisciplinary Department of Medicine, Section of Occupational Medicine, University of Bari, Bari, Italy; 6 U.O.C. Penitentiary Medicine—Department of Territorial Care, Bari Local Health Authority, Bari, Italy; 7 Department of Veterinary Medicine, University of Bari, Bari, Italy; 8 Humanitas Gavazzeni, Bergamo, Italy; 9 Department of Biotechnology, Chemistry and Pharmacy, University of Siena, Siena, Italy

**Keywords:** RNA vaccines, Viral infection

## Abstract

The SARS-CoV-2 Omicron variant has rapidly replaced the Delta variant of concern. This new variant harbors worrisome mutations on the spike protein, which are able to escape the immunity elicited by vaccination and/or natural infection. To evaluate the impact and susceptibility of different serum samples to the Omicron variant BA.1, samples from COVID-19 patients and vaccinated individuals were tested for their ability to bind and neutralize the original SARS-CoV-2 virus and the Omicron variant BA.1. COVID-19 patients show the most drastic reduction in Omicron-specific antibody response in comparison with the response to the wild-type virus. Antibodies elicited by a triple homologous/heterologous vaccination regimen or following natural SARS-CoV-2 infection combined with a two-dose vaccine course, result in highest neutralization capacity against the Omicron variant BA.1. Overall, these findings confirm that vaccination of COVID-19 survivors and booster dose to vaccinees with mRNA vaccines is the correct strategy to enhance the antibody cross-protection against Omicron variant BA.1.

## Abstract (web summary)

Antibodies elicited by a triple homologous or heterologous vaccination regimen or following natural SARS-CoV-2 infection combined with a two-dose vaccine course, result in highest neutralization capacity against the Omicron variant BA.1.

## Introduction

Since the first isolation of SARS-CoV-2 in China in January 2020, several viral variants have been detected worldwide, some of which are designated as “variants of concern” (VOCs). So far, five VOCs have been identified on the basis of one or more of the following attributes: increased transmissibility, increased virulence, increased disease severity and decreased immune protection induced by vaccination or previous infection^1,2^. The latest emerging variant, named Omicron (Pango lineage B.1.1.529), was first reported in South Africa and Botswana in November 2021^1^ and is now spreading worldwide. Omicron is the most divergent variant^3^ and is characterized by a constellation of more than 50 mutations, 30 of them on the spike (S) protein^4^. Notably, 15 mutations are located in the receptor binding domain (RBD) and some overlap with other SARS-CoV-2 variants^5–7^. The S protein plays an essential role in viral attachment, fusion, entry and transmission, and is the primary target of the current vaccines, which induce the production of neutralizing antibodies^8^. The presence of some S mutations found in other VOCs and associated with reduced neutralization activity in vaccinated subjects or previously infected individuals raises concerns regarding vaccine effectiveness and immune escape^3,5^.

To date, five vaccines, based on different technologies, have received conditional marketing authorization in Europe^9^ and are based on the S protein of the ancestral wild-type (WT) SARS-CoV-2 virus. The BNT162b2 and mRNA-1273 vaccines have been developed by using the mRNA platform technology and are manufactured by Pfizer-BioNTech and Moderna, respectively. Another two are adenovirus vectored vaccines (Ad26.COV2.S and ChAdOx1-S) manufactured by Janssen/Johnson & Johnson and AstraZeneca. The last one is a recombinant SARS-CoV-2 nanoparticle vaccine (NVX-CoV2373) designed by Novavax.

The available vaccines have been seen to offer protection against SARS-CoV-2. However, vaccines based on mRNA technology seem to be more effective at preventing symptomatic disease^10^.

The efficacy and effectiveness of COVID-19 vaccines might be influenced by several factors, such as the emergence of viral variants able to evade the immune response, the decline of antibody levels over time, and some other intrinsic host factors^11–14^. In order to maintain long-term protection and to counteract the reduced ability of available vaccines of neutralizing emerging VOCs, a third booster dose of mRNA vaccine is strongly recommended, since it has proved to confer significantly greater protection^14–17^.

This study aimed to assess the antibody-mediated immune response (both binding and neutralizing antibodies) against the SARS-CoV-2 Omicron variant (sublineage BA.1) in hospitalized COVID-19 patients and subjects who had undergone homologous or heterologous vaccination.

## Results

To evaluate the impact and susceptibility of different antibody samples to the recent Omicron variant BA.1, 189 sera from COVID-19 patients and vaccinated subjects were tested for their ability to bind and neutralize the original SARS-CoV-2 virus first detected in Wuhan, China, and the SARS-CoV-2 Omicron VOC. The serum samples were grouped into 5 different cohorts: specimens from hospitalized COVID-19 patients (*n* = 37); individuals vaccinated with 2 doses of homologous mRNA vaccine and tested negative for SARS-CoV-2 nucleocapsid (N) antibodies (*n* = 50); subjects who had received 2 doses of homologous mRNA vaccine and tested positive for SARS-CoV-2 N antibodies (indicative of previous infection) (*n* = 23); individuals who had received 3 doses of homologous mRNA vaccine (*n* = 44); and subjects who had completed a 2-dose course of adenovirus-based vaccination followed by a booster dose with an mRNA vaccine (*n* = 35) (heterologous vaccination). Binding and neutralizing activity were determined for each sample by means of an established in-house RBD ELISA or a live-virus cytopathic effect (CPE)-based microneutralization (MN) assay, respectively.

**Fig. 1:**
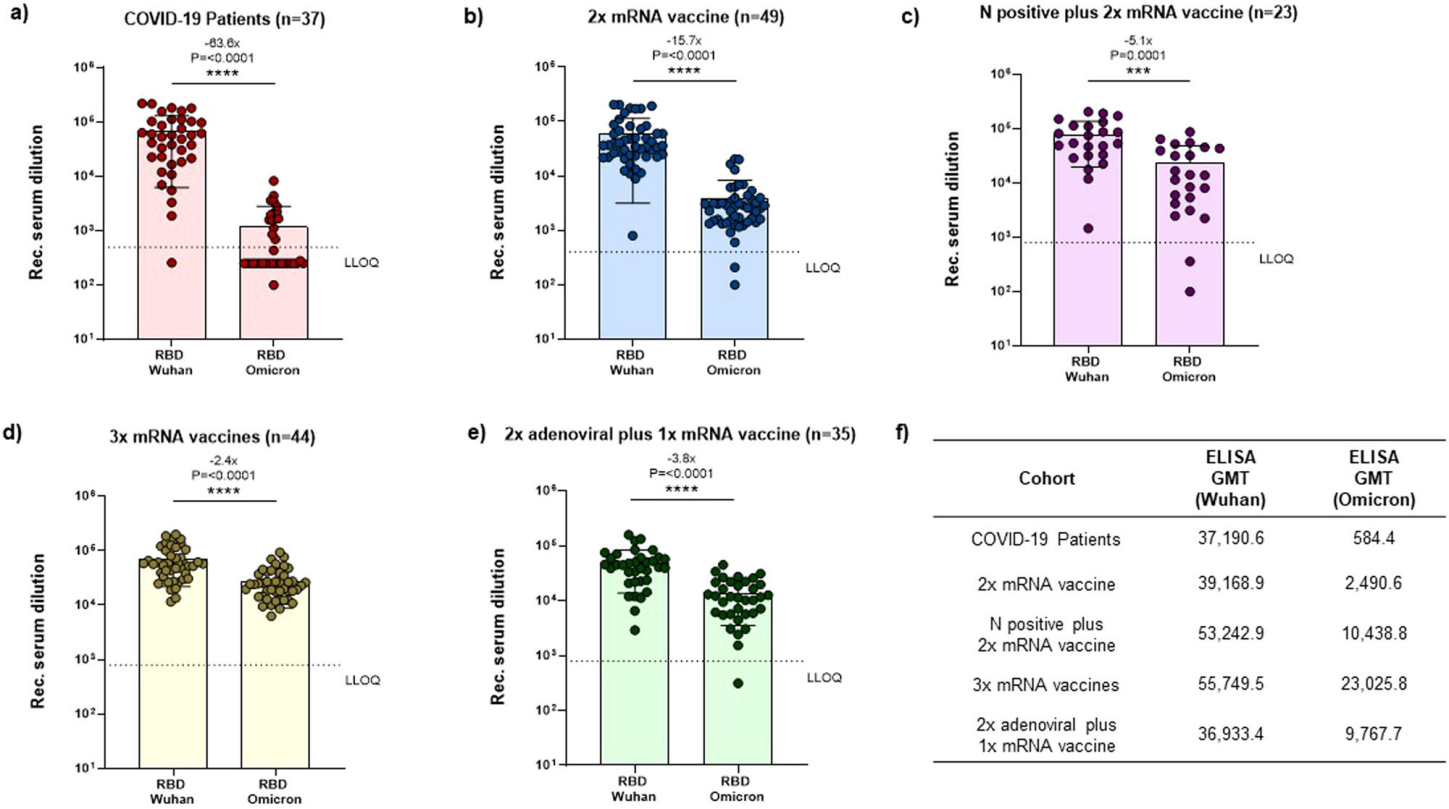
Anti-IgG ELISA binding titers against ancestral (Wuhan WT) or Omicron BA.1 RBD. **a** Hospitalized COVID-19 patients (37 subjects); **b** SARS-CoV-2-*naïve* vaccinees immunized with two doses of homologous mRNA vaccine (49 subjects); **c** previously infected subjects who had received a double dose of homologous mRNA vaccine (23 subjects); **d** vaccinees boosted with a third dose of mRNA after completion of primary double-dose vaccination with mRNA-based (homologous) vaccine (44 subjects); **e** vaccinees boosted with a third dose of mRNA after completion of primary double-dose vaccination with adenovirus-based (heterologous) vaccination (35 subjects); **f** ELISA Geometric mean titers (ELISA GMT) for each cohort and for ancestral virus and Omicron BA.1 variant. Y axis shows the reciprocal of serum dilutions (Rec. serum dilution). Data points show individual serum ELISA titers (average of two replicates). The ELISA titer is represented as the reciprocal of the highest serum dilution able to provide an absorbance value greater than the cut-off value. ELISA GMTs for each cohort are shown. Error bars indicate the GMT of the group ± standard deviation. Fold-changes in GMT are reported above histograms. *P* values were calculated by means of the Mann–Whitney U-test. Horizontal dashed line represents the Lower Limit of Quantification (LLOQ) of the assay. Different LLOQ were set according to the expected response of each cohort (COVID-19 patients LLOQ: 500; 2x mRNA vaccine LLOQ: 400; N positives plus 2x mRNA vaccines LLOQ: 800; 3x mRNA vaccine LLOQ = 800, 2x adenoviral plus 1xmRNA vaccine LLOQ: 800).

All cohorts exhibited high titers of anti-RBD IgG antibodies against the ancestral Wuhan WT virus, with the highest ELISA Geometric Mean Titer (GMT) being observed in individuals who had received 3 doses of homologous mRNA vaccine (“3x mRNA vaccine”, ELISA GMT = 55,749.5) (Fig. 1d, f). Similar titers were observed in subjects who had completed the 2-dose mRNA vaccination schedule and showed serologic evidence of previous SARS-CoV-2 infection (“N positive plus 2x mRNA vaccine”, ELISA GMT = 53,242.9) (Fig. 1c, f). Recognition of the WT-RBD was also excellent in the other three cohorts, among which GMTs were comparable (Fig. 1a, b, e). However, these GMTs were approximately 1.5-fold lower than those observed in the triple homologous mRNA vaccination group or double mRNA vaccinated subjects with N protein positivity (Fig. 1f).

On evaluating Omicron BA.1 RBD binding titers, we observed that, despite a statistically significant decrease in GMT (*p*-value ≤ 0.0001) in comparison with the WT RBD, an average of 88.36% of subjects in all groups retained their ability to recognize this antigen. Almost all vaccinees (either previously infected or not) displayed cross-recognition of the BA.1 RBD (subjects receiving 2 doses of mRNA vaccine: 95.91%; N positive plus 2x mRNA vaccine: 91.30%; 3x mRNA vaccine: 100%; 2x adenoviral plus 1xmRNA: 97.14%), whereas only 40.54% of unvaccinated COVID-19 patients showed binding activity towards the variant RBD. COVID-19 hospitalized patients exhibited the most dramatic reduction in Omicron BA.1 ELISA titers (63.6-fold decrease in GMT), followed by those who had received the 2-dose series of homologous mRNA vaccine without a booster dose (“2x mRNA vaccine”) (15.7-fold decrease in GMT) (Fig. 1a, b). Regardless of the type of vaccine, completion of a double vaccination course coupled with a previous history of infection or with a third vaccine dose was associated with the smallest reduction in Omicron BA.1 ELISA titers, with only a 2- to 5-fold decrease in GMTs in comparison with WT-RBD binding in the same groups (Fig. 1c–e). Administration of a double dose of an adenoviral vaccine followed by an mRNA booster, despite evidence of lower titers against the ancestral RBD than in the other groups, showed only approximately 2-fold lower Omicron BA.1 RBD binding titers than the 3x homologous mRNA and the N positive plus 2x mRNA vaccination groups, thus demonstrating good cross-recognition of the B.1.1.529 RBD (Fig. 1c–e). Hospitalized COVID-19 patients and mRNA double-vaccinated subjects showed 39.4- and 9.2-fold reductions, respectively, in ELISA titers against the Omicron BA.1 RBD in comparison with individuals who had received three shots of homologous mRNA vaccine (Fig. 1a, b, d).

**Fig. 2:**
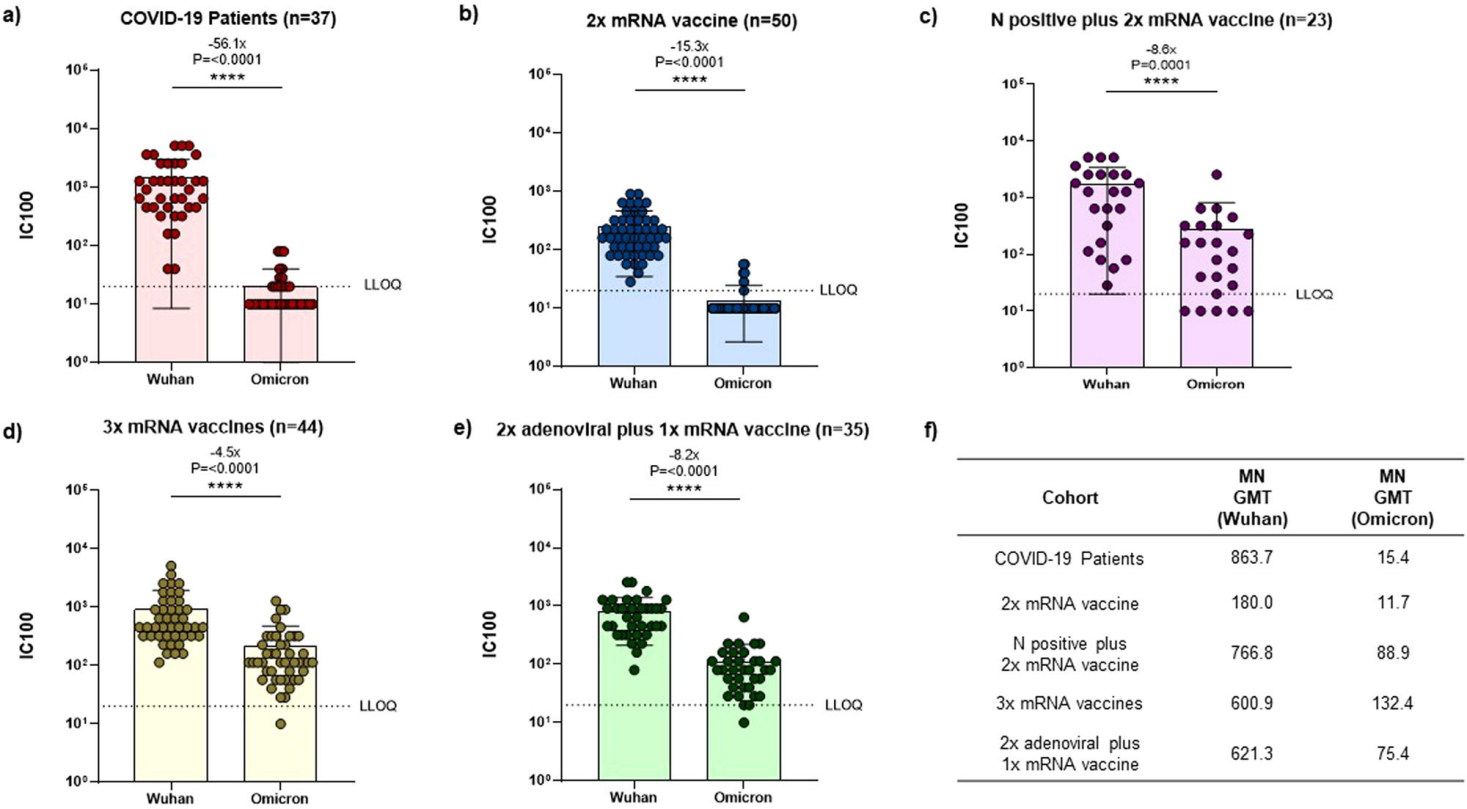
Neutralization titers against ancenstral (Wuhan WT) or Omicron BA.1 live virus. **a** Hospitalized COVID-19 patients (37 subjects); **b** SARS-CoV-2-*naïve* vaccinees immunized with two doses of homologous mRNA vaccine (49 subjects); **c** previously infected subjects who had received a double dose of homologous mRNA vaccine (23 subjects); **d** vaccinees boosted with a third dose of mRNA after completion of primary double-dose vaccination with mRNA-based (homologous) vaccine (44 subjects); **e** vaccinees boosted with a third dose of mRNA after completion of primary double-dose vaccination with adenovirus-based (heterologous) vaccination (35 subjects); **f** Neutralization (MN) Geometric Mean titers (MN GMTs) for each cohort and for ancestral virus and Omicron BA.1 variant. Data points show individual serum neutralization titers (average of two replicates). The neutralization titer is represented as the highest serum dilution able to inhibit 100% of virus-induced CPE (100% inhibitory serum dilution (IC100)). MN GMTs for each cohort are shown. Error bars indicate the GMT of the group ± standard deviation. Fold-changes in GMT are reported above histograms. *P* values were calculated by means of the Mann–Whitney U-test. Horizontal dashed line represents the Lower Limit of Quantification (LLOQ) of the assay.

We next assessed the neutralization activity against the original WT virus and the Omicron BA.1 VOC in all cohorts. In line with previous studies^6,18–24^, we observed that N-positive subjects who had received 2 mRNA vaccine doses and subjects immunized with 3 vaccine doses (whether homologous or heterologous) showed overall the highest MN geometric mean titers (MN-GMTs) (Fig. 2c–f). On evaluating neutralization activity against the WT virus, we observed high titers in all groups, with MN-GMTs ranging from 180.0 to 863.7 (Fig. 2). Indeed, the 2x mRNA vaccination group showed the lowest neutralization activity, with up to 4.8-fold lower MN-GMTs than the other groups (Fig. 2b). In line with the previous reports^6,18–21^, a drastic reduction in serum neutralization activity against the Omicron variant BA.1 was observed in all cohorts assessed. Only 35.1% (13/37) of COVID-19 hospitalized patients and 12% (6/50) of double-vaccinated individuals were able to neutralize the Omicron variant, showing a 56.1-fold (*p* < 0.0001) and 15.3-fold (*p* < 0.0001) MN-GMT reduction, respectively, in comparison with the WT MN-GMTs (Fig. 2a, b, f). Conversely, 73.9% (17/23), 97.7% (43/44) and 97.1% (34/35) of N positive plus 2x mRNA, 3x homologous mRNA and 2x adenoviral plus 1x mRNA vaccinees, respectively, showed neutralization activity against Omicron BA.1 (Fig. 2c–e). Although the majority of subjects in these latter groups retained their neutralization activity, an average 7.1-fold MN-GMT reduction was observed, with the 3x homologous mRNA vaccinees showing the smallest reduction (4.5-fold) (Fig. 2d, f).

## Discussion

The SARS-CoV-2 Omicron variant has rapidly replaced the Delta VOC in most European countries and, as anticipated by the World Health Organization, it is expected to display more than 50% seroprevalence in the European population in the coming weeks^25,26^. This new VOC harbors worrisome S mutations that are able to escape the immunity elicited by vaccine and/or natural infection.

In this study, we evaluated the extent of binding and neutralizing antibodies towards the Omicron variant BA.1 in nearly 200 serum samples collected from different cohorts of subjects, including COVID-19 patients hospitalized during the first pandemic wave and individuals who had undergone homologous and/or a heterologous vaccination. These latter individuals included 50 subjects who had received two doses of the same mRNA vaccine and negative to the N protein, 23 subjects immunized with two homologous mRNA vaccine doses who also presented anti-N protein antibodies (indicative of exposure to the natural virus), 44 subjects who had received three doses of homologous mRNA vaccine, and 35 subjects who had received a booster dose of mRNA vaccine after completion of a primary vaccination cycle (double dose) with an adenoviral vaccine.

COVID-19 patients showed the most marked reduction in Omicron BA.1 specific antibody response in comparison with the WT, resulting in the greatest drop in ELISA and MN GMTs (up to 56- and 63-fold, respectively). Indeed, most of the sera from our group of COVID-19-patients yielded an Omicron BA.1 response below the LLOQ. These results were not completely unexpected. In a previous study performed on these samples^27^, 61.9%, 88.1% and 90.5% of the samples showed a ≥ 2-fold decrease in neutralizing antibody titers against the Alpha, Gamma and Beta variants, respectively. We can speculate that the significant reduction observed was due to the fact that these patients had been naturally infected by the ancestral virus, which is antigenically different from the past VOCs and substantially divergent from the B.1.1.529 variant. Several other studies have reported a decrease or absence of neutralization capacity against VOCs in infected and/or convalescent subjects^7,28–33^, supporting the hypothesis that unvaccinated individuals exposed to SARS-CoV-2 may not be protected against current and emerging variants bearing major escape mutations, but might well still be protected from severe disease, and that cross-neutralization could be impacted by the phylogenetic distance between variants^7,29^. Natural infection, however, seems to boost the immunity elicited by vaccination considerably, suggesting that further exposure to the viral antigens may enhance protection^6,24^. Many studies have shown that Omicron-neutralizing (compared with WT-neutralizing) antibodies are higher in previously infected vaccinees, even higher than those observed in SARS-CoV-2-*naïve* subjects immunized with two mRNA vaccine doses only^6,7,29,30,34^. Our results are in line with these findings. Indeed, most of the subjects in our double-vaccinated cohort who presented signs of previous SARS-CoV-2 infection exhibited detectable neutralizing and binding antibodies against the Omicron variant BA.1; moreover, they displayed a smaller reduction in ELISA and MN GMTs (in comparison with GMTs against the ancestral virus) than the cohort of *naïve* double-vaccinated individuals.

On the other hand, consistently with the previous reports^6,30^, our results highlight the ability of the Omicron variant BA.1 to escape the immune response elicited by two vaccine doses. Other studies have reported a significantly lower neutralizing ability of two doses of mRNA vaccine against Omicron and the other VOCs, such as Beta and Delta^6,35–37^. Notably, our study showed that, despite displaying relatively good antibody binding to the Omicron BA.1 RBD (4.3-fold higher titers than those of COVID-19 patients), sera from SARS-CoV-2-*naïve* double-vaccinated subjects completely lost their ability to neutralize the live VOC. By contrast, the other groups of vaccinees showed similar trends in ELISA and neutralization titers. The cohort of *naïve* subjects who had received a double vaccine dose also displayed lower neutralization activity than the other vaccinees (including those previously infected) towards the ancestral virus. This suggests that administering a booster dose of ancestral S protein (in the context of either a homologous or a heterologous triple vaccination strategy) or a natural infection combined with a double ancestral S dose can yield superior humoral immunity both against the original and a heavily mutated SARS-CoV-2 virus.

In our study, the immune response against the Omicron variant BA.1 strongly benefited from a booster dose with an mRNA vaccine, after either a homologous or a heterologous (adenoviral vaccine) double-dose vaccination regimen. Indeed, the cohort of triple-vaccinated subjects (3 mRNA doses) showed the least reduction in antibodies, both neutralizing and binding, against the B.1.1.529 lineage. These findings are consistent with previous observations that an additional dose administered after completion of a primary vaccination series induces the most potent and cross-reactive antibody response^6,7,30,35–37^. The explanation might be that the third dose boosts the immune system, allowing cross-neutralizing responses against the new variant, either through further affinity maturation of existing antibodies or by targeting new epitopes shared with the other variants^6^.

The last cohort in our study consisted of subjects who had received heterologous prime-boost (two doses of adenoviral vaccine and one dose of mRNA vaccine). This vaccine combination conferred some degree of cross-neutralization of the Omicron variant BA.1, resulting in approximately 4-fold and 8-fold reductions in ELISA and MN GMTs, respectively (relative to the WT); this is in agreement with a previous study^33^. These reductions were considerably lower than in COVID-19 patients or double-vaccinated only subjects, and similar to those observed in previously infected vaccinees or triple-vaccinated subjects who had received a homologous mRNA vaccine.

According to our study, the third vaccine dose did not substantially enhance neutralization titers against the ancestral virus in comparison with infection-only or previous infection coupled with a double vaccination course; however, the third dose was associated with an increase in the neutralization capacity against the Omicron variant BA.1: 7 to 10-fold higher than that seen in COVID-19 patients or SARS-CoV-2-*naïve* double-vaccinees. Indeed, individuals with signs of previous SARS-CoV-2 infection who had completed the two-dose mRNA vaccination series showed an increase in Omicron-neutralizing antibody titers that was similar to those seen in the triple-vaccinated subjects on either a homologous or a heterologous prime-boost regimen. These findings further support the conviction that a prime-boost regimen with the ancestral SARS-CoV-2 S (but not natural infection alone) or hybrid immunity can elicit an antibody response (even if sub-optimal) against the B.1.1.529 lineage, the extent and potency of which seems to increase with the number of S-protein exposures.

A key strength of this study was the use of an MN assay with authentic live SARS-CoV-2 viruses and not a surrogate neutralization assay. In addition, the long assay incubation time (three or four days) of the virus-sample mixture in cell cultures can enable us to identify more precisely the antibody titer that could best correlate with the real protection, since this titer is based on the complete inhibition of the CPE in the cell monolayer.

However, this study has some limitations. Serum samples from COVID-19 patients were collected during the first pandemic wave and may not be completely representative of the currently infected population. The number of enrolled subjects was relatively small (*n* = 189); however, all the cohorts considered reflect the different situations in the general population. The timing of post-vaccination blood withdrawal was not perfectly matched between subjects who had undergone heterologous vaccination and the other cohorts. Gender distribution is not balanced, 88,16% of subjects included in the study are male, while only 11,84% are female. Furthermore, we did not evaluate other branches of immunity, such as T cell responses, which could contribute to protection even when neutralizing antibodies are absent or reduced. Lastly, we did not evaluate the antibody responses against currently circulating subvariants such as BA.2.12.1 and BA.4, or the BA.5, which is quickly becoming the dominant SARS-CoV-2 strain worldwide. Although the three-doses vaccination regimen with the currently available vaccines seems to provide acceptable neutralizing-antibody titers against these subvariants, they also display increased evasion of neutralizing antibodies compared to BA.1 and BA.2^38,39^.

Overall, our results confirm previously reported evidence that the potency of naturally induced or vaccine-elicited neutralizing antibodies against the SARS-CoV-2 Omicron variant BA.1 is very low or even absent, and that a third dose of mRNA vaccine broadens the humoral immune response and increases neutralizing and binding antibodies against the B.1.1.529 lineage. Antibodies produced following a triple homologous/heterologous vaccination regimen or following natural SARS-CoV-2 infection plus a two-dose vaccine course, result in greater neutralization of the Omicron variant BA.1 than the administration of two doses of homologous vaccine in SARS-CoV-2-*naïve* subjects; in agreement with previous reports, we conclude that natural infection alone or a double vaccination regimen in SARS-COV-2-*naïve* subjects cannot counteract Omicron infection. An mRNA booster dose, in the context of either a homologous or a heterologous vaccination regimen, might therefore be necessary to achieve neutralizing antibody titers against the live Omicron variants.

In addition, emerging sub-lineages have posed concern due to their higher escape neutralization suggesting that the Omicron variant has continued to evolve towards increasing its ability to evade the antibody response^39,40^. Although virus neutralization seems to be lower compared that against the BA.1 variant, vaccinated groups have demonstrated an increased neutralization capacity (five-fold higher) against these emerging variants higher than unvaccinated group^41^.

Altogether, the results of this study support the current strategy of administering an mRNA vaccine administration and booster to enhance antibody-based cross-protection and protect against emerging Omicron variants.

## Methods

### Study population

For the aim of the study, serum samples were grouped into 5 different cohorts.

Thirty-seven (37) serum samples from COVID-19 patients hospitalized at Humanitas Gavazzeni (Bergamo, Italy) during the first pandemic wave (March-May 2020) were included in the present study. Subjects’ characteristics and study procedures have been described in detail elsewhere^42^. For the purpose of the present study, only samples collected on day 6 after hospitalization were selected, since they showed the highest neutralizing antibody titers against the 2019-nCov/Italy-INMI1 strain (WT virus)^42^. The study was conducted in accordance with the Declaration of Helsinki and samples have been fully anonymized before testing. This study was approved by the Ethics Committee of the University of Siena (approval number 17373) and by the Ethics Committee of Humanitas Gavazzeni (approval number 236).

Fifty (50) serum samples were collected at the Bari correctional facility (Apulia, Italy) a mean of 21 days after the 2^nd^ dose of one of the two mRNA vaccines approved (mRNA −1273 and BNT162b2). These samples showed to be negative when tested for the N protein by means of a commercial ELISA kit (IDScreenSARS-CoV-2 Double Antigen Multi-species ELISA, ID.vet, Grabels, France).

Twenty-three (23) serum samples were collected at the Bari correctional facility (Apulia, Italy) a mean of 20 days after the 2^nd^ dose of mRNA vaccine (mRNA −1273 and BNT162b2). These samples showed positivity to antibodies against the N protein when tested by means of a commercial ELISA kit (IDScreenSARS-CoV-2 Double Antigen Multi-species ELISA, ID.vet, Grabels, France).

Forty-four (44) serum samples were collected at the Bari correctional facility (Apulia, Italy) a mean of 21 days after the 3^rd^ mRNA vaccine dose (mRNA −1273 and BNT162b2).

Thirty-five (35) serum samples were collected from employees of the University of Bari a mean of 42 days after the 3^rd^ dose of mRNA vaccine. These subjects received two doses of adenoviral vaccine and a booster dose (3^rd^ dose) with mRNA vaccine (mRNA −1273 or BNT162b2).

The research protocol was approved by the Ethics Committee of the University Hospital of Bari (n. 6955, prot. N. 0067544–02082021). The serum survey was conducted in accordance with ethical principles (Declaration of Helsinki), and written informed consent was obtained from all the participants.

Serum samples were tested in duplicate for each assay.

### Cells and viruses

African green monkey kidney Vero E6 cells (American Type Culture Collection [ATCC] #CRL-1586/Vero C1008) were cultivated in Dulbecco’s Modified Eagle’s Medium high glucose (DMEM) (Euroclone, Pero, Milan) supplemented with 2 mM L-Glutamine (Euroclone, Pero, Milan), 100 U/mL of penicillin - 100 µg/mL streptomycin (P/S Gibco, Life Technologies) (complete DMEM) and 10% Fetal Bovine Serum (FBS) (Euroclone, Pero, Milan). Cells were maintained at 37 °C, in a humified 5% CO_2_ environment, and passaged every 3-4 days. 18-24 h before execution of the MN assay, plates were seeded with 100 μL/well of Vero E6 cells (1.5×10^5^ cell/mL) diluted in complete DMEM supplemented with 2% FBS (DMEM 2% FBS), and incubated at 37 °C, 5% CO_2_ until use.

Authentic WT SARS CoV-2 2019 (2019-nCov/Italy-INMI1 strain) virus was purchased from the European Virus Archive goes Global (EVAg, Spallanzani Institute, Rome). The live Omicron SARS-CoV-2 variant, sublineage BA.1, was kindly provided by Prof. Piet Maes, NRC UZ/KU Leuven (Leuven, Belgium). Omicron sequence was deposited on GISAID with the following ID: EPI_ISL_6794907.

Viral propagation was performed in 175 cm^2^ tissue-culture flasks pre-seeded with 50 mL of Vero E6 cells (1×10^6^ cells/mL) diluted in DMEM 10% FBS. After 18–20-hour incubation at 37 °C, 5% CO_2_, flasks were washed twice with sterile Dulbecco’s phosphate buffered saline (DPBS) and then inoculated with the SARS-CoV-2 virus at a multiplicity of infection (MOI) of 0.001. The sub-confluent cell monolayer was incubated with the virus for 1 h at 37 °C, 5% CO_2_, then flasks were filled with 50 mL of DMEM 2% FBS and incubated at 37 °C, 5% CO_2_. Cells were monitored daily until manifestation of 80-90% CPE. Supernatants of the infected cultures were then harvested, centrifuged at 469 × g for 5 min at 4 °C to remove cell debris, aliquoted and stored at −80 °C.

The propagated viral stocks were titrated in 96-well plates previously seeded overnight with VERO E6 cells. 10-fold serial dilution of virus (10^−1^ to 10^−11^) were incubated with the cells and checked for signs of CPE for a total of 72 h (WT strain) or 96 h (Omicron variant). The viral titer was calculated by using the 50% tissue culture infectious dose per mL (TCID50/mL) as the endpoint and defined as the reciprocal of the highest virus dilution yielding at least 50% CPE in the inoculated wells, according to the Reed and Munch formula^43^.

### Microneutralization assay with authentic SARS-CoV-2 viruses

For the MN assay, 2-fold serial dilutions of the samples (starting dilution 1:20) were prepared in duplicate in DMEM 2% FBS and added to two different 96-well plates. The plates were then incubated for 1 h at 37 °C with a standard concentration of the virus (sample-virus ratio 1:1)^44^. Following incubation, the virus-sample mixture was then added to sub-confluent Vero E6 cells to assess whether the virus had retained its infectious capacity. After 72 h (WT strain) or 96 h (Omicron variant) cells were inspected for signs of CPE. The highest sample dilution able to completely inhibit viral growth, in terms of CPE, was regarded as the neutralization titer.

The test was executed in one session on the same day for each strain. A cell-only and a virus-only control were added to each row of each plate to monitor the status of the cell monolayer and the virus itself within each plate. A negative control sample (negative plasma code 20/142 from WHO NIBSC panel 20/268) and a positive control sample (pooled plasma high positive in terms of anti-SARS-CoV-2 immunoglobulins, code 20/150 from WHO NIBSC panel 20/268) were included, in duplicate, in a separate plate as a control of the assay session. Parallel titrations of the viruses were performed in 96-well plates containing sub-confluent Vero E6 cells, as previously described, to monitor the viral titer.

### In-house enzyme-linked immunosorbent assay (ELISA)

IgG determination in human serum samples was performed by using an in-house ELISA RBD assay. 96-well ELISA plates were coated with 1 µg/mL of purified recombinant Wuhan SARS-CoV-2 Spike-RBD protein (Arg319-Phe541) (Sino Biological, Beijing, China) or B.1.1.529 RBD (Arg319-Phe541) (Sino Biological, Beijing, China), both expressed and purified from HEK 293 cells. Plates were incubated at 4 °C overnight and washed with 300 µL/well of Tris Buffered Saline (TBS)-0.05% Tween 20 (T-TBS), then blocked for 1 h at 37 °C with a solution of T-TBS containing 5% of Non-Fat Dry Milk (NFDM, Euroclone, Pero, Italy). Serum samples were serially diluted in 2-fold dilutions in 5% NFDM/T-TBS. Plates were washed three times with T-TBS, then 100 μL of each serial dilution was added to the plates and incubated for 1 h at 37 °C. The plates were then washed three times and 100 µL of Goat anti-Human IgG-Fc Horse Radish Peroxidase (HRP)-conjugated antibody (Bethyl Laboratories, Montgomery, USA) diluted 1:100,000 in 5% NFDM/T-TBS was added to each well. The plates were then incubated at 37 °C for 30 min and, after three washing steps, 100 μL/well of 3,3′,5,5′ -Tetramethylbenzidine (TMB) substrate (Bethyl Laboratories, Montgomery, USA) was added and incubated in the dark at room temperature for 20 min. The reaction was stopped by adding 100 μL of hydrochloric acid solution 0.5 M (Fisher Chemical, Milan, Italy) and read within 20 min at 450 nm with a SpectraMax ELISA plate (Medical Device) reader. A cut-off value was defined as 3 times the average of optical density OD values from blank wells (background: no addition of analyte). Samples with ODs below the cut-off value at the lowest dilution were assigned a negative value, while samples with ODs above the cut-off value at the lowest dilution were deemed positive^45^. Based on the expected antibody response, a different lower limit of quantification (LLOQ) was used for each cohort.

### Statistics and reproducibility

Data analysis was performed by means of GraphPad Prism Version 5 and Microsoft Excel 2019. Data were log transformed and then the non-parametric Mann–Whitney U-test analysis was performed to evaluate statistical significance between the 5 different cohorts analysed in this study. Statistical significance was shown as *P ≤ 0.05, **P ≤ 0.01, ***P ≤ 0.001, ****P ≤ 0.0001.

### Reporting summary

Further information on research design is available in the Nature Research Reporting Summary linked to this article.

## Supplementary information

Description of Additional Supplementary Files

Supplementary Data 1

Supplementary Data 2

Reporting Summary

## Data Availability

The authors declare that all data supporting the findings of this study are available. within the supplementary information files (Supplementary Data 1 and 2).
