# Supplementary material for: Immune response to SARS-CoV-2 Omicron variant in patients and vaccinees following homologous and heterologous vaccinations
Source: Commun Biol. 2022 Sep 2;5:903. doi: 10.1038/s42003-022-03849-0 (PMC9439265; doi:10.1038/s42003-022-03849-0)
Supplement: Supplementary file 1 — Description of Additional Supplementary Files [file 42003_2022_3849_MOESM1_ESM.pdf]

Description of Additional Supplementary Files

File name: Supplementary Data 1

Description: Raw data from Fig. 1a, 1b, 1c, 1d, 1e and 1f.

File name: Supplementary Data 2

Description: Raw data from Fig. 2a, 2b, 2c, 2d, 2e and 2f.
