# Supplementary material for: Immune response to SARS-CoV-2 Omicron variant in patients and vaccinees following homologous and heterologous vaccinations
Source: Commun Biol. 2022 Sep 2;5:903. doi: 10.1038/s42003-022-03849-0 (PMC9439265; doi:10.1038/s42003-022-03849-0)
Supplement: Supplementary file 4 — Reporting Summary [file 42003_2022_3849_MOESM4_ESM.pdf]

nature research

Corresponding author(s): Claudia Maria Trombetta

Last updated by author(s): Aug 1, 2022

Reporting Summary

Nature Research wishes to improve the reproducibility of the work that we publish. This form provides structure for consistency and transparency in reporting. For further information on Nature Research policies, see our Editorial Policies and the Editorial Policy Checklist.

Statistics

For all statistical analyses, confirm that the following items are present in the figure legend, table legend, main text, or Methods section.

n/a	Confirmed	
☐	☒	The exact sample size (*n*) for each experimental group/condition, given as a discrete number and unit of measurement
☐	☒	A statement on whether measurements were taken from distinct samples or whether the same sample was measured repeatedly
☐	☒	The statistical test(s) used AND whether they are one- or two-sided<br>*Only common tests should be described solely by name; describe more complex techniques in the Methods section.*
☒	☐	A description of all covariates tested
☒	☐	A description of any assumptions or corrections, such as tests of normality and adjustment for multiple comparisons
☒	☐	A full description of the statistical parameters including central tendency (e.g. means) or other basic estimates (e.g. regression coefficient) AND variation (e.g. standard deviation) or associated estimates of uncertainty (e.g. confidence intervals)
☐	☒	For null hypothesis testing, the test statistic (e.g. F, t, r) with confidence intervals, effect sizes, degrees of freedom and P value noted<br>*Give P values as exact values whenever suitable.*
☒	☐	For Bayesian analysis, information on the choice of priors and Markov chain Monte Carlo settings
☒	☐	For hierarchical and complex designs, identification of the appropriate level for tests and full reporting of outcomes
☒	☐	Estimates of effect sizes (e.g. Cohen's d, Pearson's r), indicating how they were calculated

Our web collection on statistics for biologists contains articles on many of the points above.

Software and code

Policy information about availability of computer code

Data collection	*Provide a description of all commercial, open source and custom code used to collect the data in this study, specifying the version used OR state that no software was used.*
Data analysis	GraphPad Prism Version 5 was used to perform statistical analyses.

For manuscripts utilizing custom algorithms or software that are central to the research but not yet described in published literature, software must be made available to editors and reviewers. We strongly encourage code deposition in a community repository (e.g. GitHub). See the Nature Research guidelines for submitting code & software for further information.

Data

Policy information about availability of data

All manuscripts must include a data availability statement. This statement should provide the following information, where applicable:

- Accession codes, unique identifiers, or web links for publicly available datasets
- A list of figures that have associated raw data
- A description of any restrictions on data availability

Source data are provided with this paper. All data supporting the findings in this study are available within the article or can be obtained from the corresponding author upon request.

Field-specific reporting

Please select the one below that is the best fit for your research. If you are not sure, read the appropriate sections before making your selection.

☒ Life sciences ☐ Behavioural & social sciences ☐ Ecological, evolutionary & environmental sciences

For a reference copy of the document with all sections, see nature.com/documents/nr-reporting-summary-flat.pdf

Life sciences study design

All studies must disclose on these points even when the disclosure is negative.

Sample size	A total of 189 samples were analyzed in this study and grouped into 5 different cohorts. We did not use statistical methods to predetermine sample size. Sample size was based on samples' availability.
Data exclusions	No data was excluded.
Replication	All experiments were performed in technical duplicates as indicated in the methods section.
Randomization	The experiments were not randomized and all available samples were tested. The authors aimed to specifically assess the antibody response against the SARS-CoV-2 Omicron variant in hospitalized COVID-19 patients and subjects who had undergone homologous or heterologous vaccination.
Blinding	The investigators were not blinded during group allocation, data collection and analyses.

Reporting for specific materials, systems and methods

We require information from authors about some types of materials, experimental systems and methods used in many studies. Here, indicate whether each material, system or method listed is relevant to your study. If you are not sure if a list item applies to your research, read the appropriate section before selecting a response.

Materials & experimental systems

n/a	Involved in the study	
☐	☒	Antibodies
☒	☐	Eukaryotic cell lines
☒	☐	Palaeontology and archaeology
☒	☐	Animals and other organisms
☐	☒	Human research participants
☒	☐	Clinical data
☒	☐	Dual use research of concern

Methods

n/a	Involved in the study	
☒	☐	ChIP-seq
☒	☐	Flow cytometry
☒	☐	MRI-based neuroimaging

Antibodies

Antibodies used	Goat anti-Human IgG-Fc Fragment Antibody HRP Conjugated Bethyl Laboratories® Catalog # A80-104P.
Validation	*Describe the validation of each primary antibody for the species and application, noting any validation statements on the manufacturer's website, relevant citations, antibody profiles in online databases, or data provided in the manuscript.*

Human research participants

Policy information about studies involving human research participants

Population characteristics	This work results from a collaboration with Humanitas Gavazzeni (Bergamo, Lombardy, Italy), the Bari correctional facility (Apulia, Italy) and the University of Bari (Apulia, Italy). All data relevant to enrolled subjects are reported in this study. Subjects elegible for this study were previously infected by SARS-CoV-2 or vaccinated with COVID-19 vaccines.
Recruitment	Subjects involved in this study were voluntary recruited in compliance with Italian law on Ethics. The authors do not see any potential bias in the generation or interpretation of the data reported in this study.
Ethics oversight	The study was approved by the Ethics Committee of the University of Siena (approval number 17373), by the Ethics Committee of Humanitas Gavazzeni (approval number 236) and by the Ethics Committee of the University Hospital of Bari (n. 6955, prot. N. 0067544–02082021).<br>This study was unblinded and not randomized. No statistical methods were used to predetermine sample size.

Note that full information on the approval of the study protocol must also be provided in the manuscript.
